# Hijacked highway: A rare case of basal cell carcinoma encasing a cranio-peritoneal shunt

**DOI:** 10.1016/j.jpra.2025.12.017

**Published:** 2025-12-18

**Authors:** Lisa Davenport, Teresa Y. Liew, Juanita Ling

**Affiliations:** a Department of Plastic and Reconstructive Surgery, Cairns Base Hospital, Queensland, Australia; b Department of Plastic and Reconstructive Surgery, Townsville University Hospital, Queensland, Australia

**Keywords:** Skin cancer, Basal cell carcinoma, Metastatic, Ventriculoperitoneal shunt, Case report

## Abstract

**Background:**

Metastatic basal cell carcinomas (BCC) are rare. In event of metastasis, BCCs are most likely to spread to the lymph nodes, lungs, bones, and skin. BCC spreading along implanted devices has not been previously documented.

**Case presentation:**

We report a case of a 48-year old man with a head and neck cutaneous BCC involving a cranio-peritoneal shunt, which holds the potential risk of tumor dissemination through this low-resistance pathway. He was followed up over his lifetime to determine if he developed cranial or peritoneal dissemination.

**Management and outcomes:**

A multi-disciplinary team approach was undertaken, a joint case between plastic surgery and neurosurgery was required to secure the shunt and complete a wide local excision. Post-operatively, the patient had adjuvant radiotherapy. The metastatic spread in this case followed the usual pattern of BCC metastasis. The patient developed lung nodules and bone metastases, and died 23 months post-operatively. He did not develop cranial or peritoneal metastases. In this case, the involvement of the shunt did not alter the expected pattern of metastatic spread of BCC.

**Conclusion:**

There is a limited number of case studies that describe implantable devices as a conduit for tumour dissemination.The risk of dissemination did not occur in this case. The long-term follow-up of this case contributes to the literature on decision making in the management of head and neck tumors involving shunts.

## Introduction

Cutaneous carcinoma involving implanted synthetic material is very rare. There has only been one other reported cutaneous malignancy involving a ventriculoperitoneal shunt (VPS).^1^ This unusual case of a BCC in the head and neck region encasing a VPS underscores the currently undefined risk of tumor dissemination posed by cutaneous malignancies involving implanted synthetic medical devices and raises important considerations for the surgical management and clinical surveillance of an otherwise straightforward BCC excision.

## Case presentation

A 48-year-old Caucasian male was referred from a regional center with a new rapidly growing cutaneous BCC on his right posterior neck encasing his cranio-peritoneal shunt. He had a background of cerebral palsy with left-sided hemiplegia, and lived in a high care residence. A VPS was inserted in childhood for hydrocephalus that had subsequently migrated to a subdural position. He had a history of multiple non-melanoma skin cancers, the most significant being an large BCC with perineural invasion (PNI) on the posterior scalp that was excised then completed adjuvant radiotherapy 40Gy/10#.

**Figure 1:**
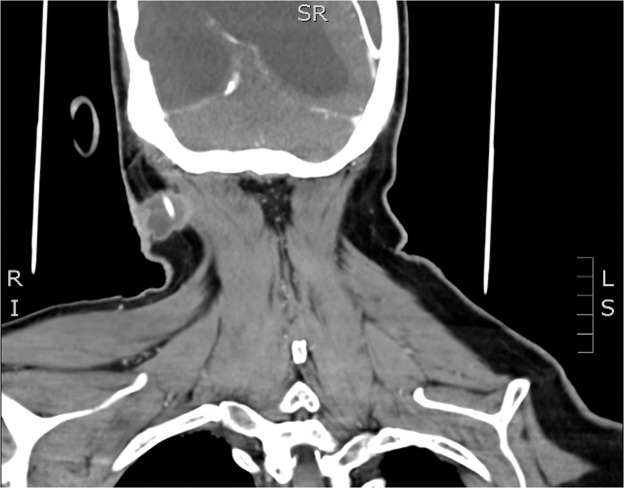
Pre-operative CT demonstrating location of cutaneous lesion encasing shunt catheter, adhering to skeletal muscles.

Examination revealed a large ulcerative cutaneous lesion at the posterior triangle of the right neck. It was tender and tethered to underlying structures, with a segment of the VPS catheter just visible at the base of the ulcer. Computed tomography (CT) demonstrated a 24 × 24 mm lesion adherent to the sternocleidomastoid and right posterior occipital neck skeletal muscles. The shunt catheter transversed through and was encased by the lesion. There was a level of fluid along the shunt in the posterior occipital region, and a 16 × 11 mm pathological lymph node deep to the sternocleidomastoid (Figure 1). No other regional or distant disease was present.

The patient was discussed in a multidisciplinary meeting. The consensus was to involve plastic surgery and neurosurgery teams to secure the shunt before a wide local excision.

**Figure 2:**
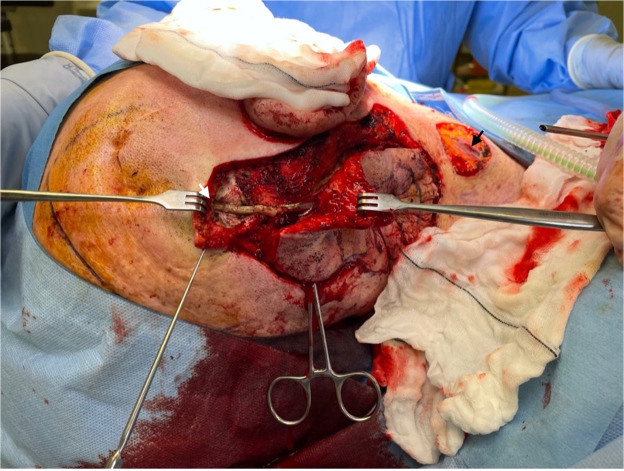
Intraoperative clinical photography of the right neck with the resultant defect. Proximal end of VPS amputated (white arrow) and distal end of VPS (black arrow) amputated beyond tumor margins.

**Figure 3:**
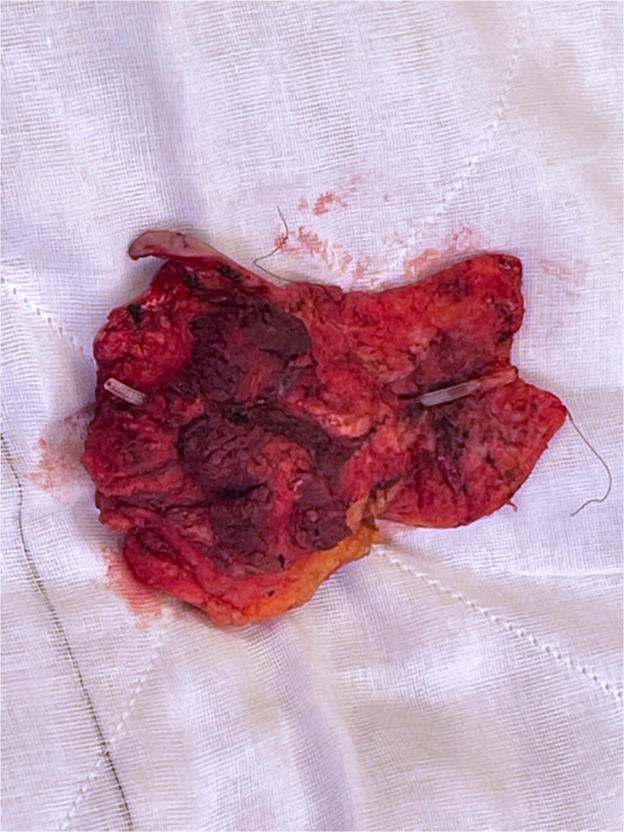
Resected specimen with shunt catheter on view.

Intraoperatively, the neurosurgery team divided the shunt catheter proximally and distally. As the catheter was old and friable, attempts to extract the cranial and peritoneal ends were unsuccessful and these were therefore left in-situ after amputation of the catheter well beyond the tumor margins (Figure 2). There was no CSF fluid found within the catheter. The Neurosurgery team elected not to replace the VPS, as the previous one had been non-functional. The plastic surgery team then resected the lesion en bloc with the amputated shunt catheter (Figure 3). The excision included skin, subcutaneous tissue, part of the sternocleidomastoid with the pathological lymph node, splenius capitus, part of levator scapulae, and the upper trapezius down to the level of prevertebral fascia. The resulting defect was reconstructed with a split thickness skin graft.

**Figure 4:**
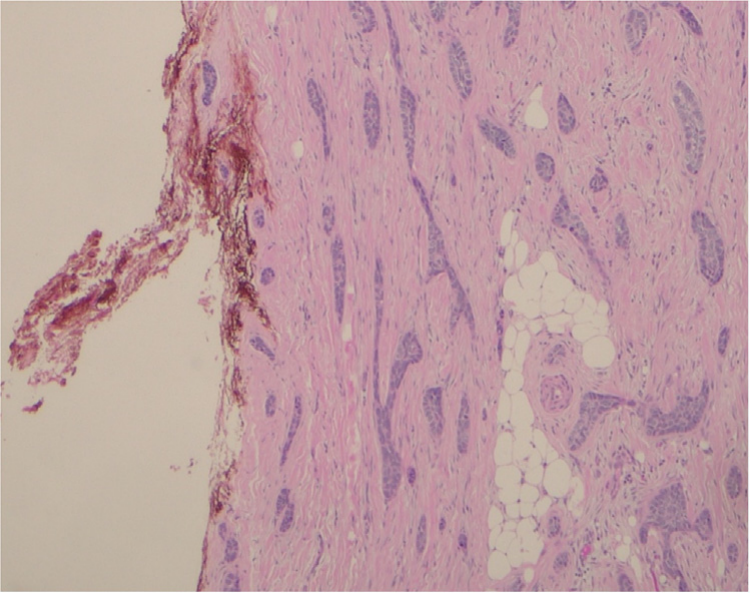
Histology slide demonstrating BCC abutting the shunt catheter (margin marked red).

Histology confirmed a 50 mm infiltrative BCC invading into skeletal muscle encasing the shunt catheter, with a depth of invasion of 20 mm. Multiple PNI involving large nerves measuring up to 0.5 mm were present. The peripheral margins were clear, but the deep margin was involved around the shunt (Figure 4). The patient had no change neurologically and the post-operative course was uneventful. He completed adjuvant radiotherapy (50Gy/20#) without complications.

Unfortunately, 14 months post-operatively, the patient developed FDG-avid lung nodules and bony metastases. Biopsy of the right ischial tuberosity demonstrated carcinoma with basaloid features, with positive pancytokeratin (AE1/AE3), GATA3, p40, and BerEP4 immunohistochemical staining. The patient was unsuitable for systemic therapy due to his comorbidities. His metastatic BCC continued to progress over the next 9 months, resulting in pathological fractures of his right ischial tuberosity requiring intramedullary nail, pathological rib fractures and lung metastases impacting breathing, and significant associated pain requiring opioid analgesia.

He died at age 50 from pneumonia in the context of splinted breathing from his pathological rib fractures.

## Discussion

Basal cell carcinoma (BCC) is the most common skin malignancy in Australia, accounting for 70 % of non-melanoma skin cancers. BCC tumorigenesis due to UV damage has the highest mutational load of any malignancy. It is well established that alteration in the sonic hedgehog cellular pathway leads to downstream effectors that promote the development of BCCs. Whilst BCCs are common, metastasis is rare and estimated to be <1 %.^2^ Understanding the genetic events that lead to low rates of dissemination and metastatic colonisation to regional and distant sites is based on limited case series due to low number of cases.^3^ Metastasis is often associated with aggressive features such as squamous differentiation, infiltrating subtypes, and perineural invasion (PNI).^4^ In the event of metastasis, BCCs are most likely to spread to lymph nodes, the lungs, bones, and skin.^2^^,^^4^ Survival times for metastatic BCC has been reported between 2 years for distant metastasis, and over 7 years for regional metastasis.^5^

VPS are often inserted to manage raised intraventricular pressures and hydrocephalus, allowing drainage of excess cerebrospinal fluid (CSF) from the brain to the peritoneal cavity. In the context of tumor extension and seeding, it can act as low-resistance pathway for tumor spread. This phenomenon has been described in various central nervous system malignancies, leading to intra-abdominal metastases^6^; and even reported in a retrograde fashion from peritoneum to the central nervous system.^7^

The primary consideration in this unique case was the theoretical possibility of VPS being a conduit for tumor dissemination. The actual risk of this occurring, however, is unknown, as this is only the second documented case.^1^ Tumor seeding along implanted tubing has been documented in other malignancies such as cutaneous squamous cell carcinoma, melanoma, mesotheliomas, central nervous system, as well as gastrointestinal malignancies.^6^, ^7^, ^8^, ^9^, ^10^

The multi-disciplinary approach was important for both pre-operative planning and post-operative management due to the unknown potential for tumor spread with shunt involvement and high-risk features of the BCC.

Pre-operative considerations included accounting for the initial aggressive features of the lesion—rapidly growing, invading skeletal muscle, and presence of a pathological lymph node, consideration of measures to prevent tract seeding, and technical management of shunt removal. The lesion was widely and deeply excised to avoid involved margins. The deep margin was unfortunately involved around the shunt, which could be avoided in the future with an intraoperative frozen section.

As the CT demonstrated fluid along the shunt tract in the posterior occipital region, meticulous care was taken during the dissection to ensure the shunt was secured both distally and proximally before excision. Since the shunt could not be extracted, it was amputated well beyond the tumor excision margins. The pieces of subdural and peritoneal tubing that were left in situ posed a foreign body infection risk, that did not eventuate. If CSF was present within the shunt, this would have been sent for cytology to exclude dissemination.

Post-operatively, routine surveillance imaging of the abdomen was deemed unsuitable due to the patient’s comorbidities. However, an incidental CT performed 20 months post-operatively for an unrelated acute presentation showed no evidence of intra-abdominal metastasis or CNS dissemination. The eventual metastatic spread of BCC was in the expected common metastatic sites.

## Conclusion

Previous literature has suggested that tumors involving implanted synthetic medical devices can act as a conduit for tumor seeding. The long-term follow-up of this case contributes to the literature on decision-making in the excision of head and neck cutaneous malignancies with VPS involvement. This case had an expected pattern of metastatic spread with the shunt not altering the pattern of metastatic spread.

## Funding

None.

## Patient consent

Written consent for the use of images in this paper was obtained from the patient’s next-of-kin.

## Ethical approval

Not required.

## Declaration of competing interest

The authors have no conflicts of interest to disclose.
